# A Novel Platform Featuring Nanomagnetic Ligand Fishing Based on Fixed-Orientation Immobilized Magnetic Beads for Screening Potential Cyclooxygenase-2 Inhibitors from *Panax notoginseng* Leaves

**DOI:** 10.3390/molecules29235801

**Published:** 2024-12-09

**Authors:** Fan Zhang, Fan Sun, Lequan Yu, Fei Li, Lixia Liu, Xiaoyan Cao, Yi Zhang, Lijie Wu

**Affiliations:** 1 College of Chinese Materia Medica, Tianjin University of Traditional Chinese Medicine, 10 Poyanghu Road, West Area, Tuanbo New Town, Jinghai District, Tianjin 301617, China; 18338595752@163.com (F.Z.); zhwwxzh@tjutcm.edu.cn (Y.Z.); 2 Tianjin Key Laboratory of TCM Chemistry and Analysis, Tianjin University of Traditional Chinese Medicine, 10 Poyanghu Road, West Area, Tuanbo New Town, Jinghai District, Tianjin 301617, China; sf18435165322@163.com (F.S.); 18822575726@163.com (L.Y.); lf18641423285@163.com (F.L.); 15689081560@163.com (L.L.); cxysssl@163.com (X.C.)

**Keywords:** ligand fishing, magnetic nanoparticles, immobilized enzymes, COX-2 inhibitors, *Panax notoginseng* leaves

## Abstract

A novel screening platform based on an Fe_3_O_4_@C@PDA-Ni^2+^@COX-2 ligand fishing combination with high-performance liquid chromatography–mass spectrometry was first designed, synthesized, and employed to screen and identify COX-2 inhibitors from *Panax notoginseng* leaves. The obtained magnetic nanoparticles exhibit outstanding preconcentration ability that allows for controlling the enzyme orientation to avoid enzyme active site blocking, conformational changes, or denaturing during immobilization. The as-prepared Fe_3_O_4_@C@PDA-Ni^2+^@COX-2 composite was carefully characterized by scanning electron microscopy (SEM), transmission electron microscopy (TEM), Fourier-transform infrared spectrometry (FT-IR), Xray powder diffraction (XRD), thermal gravimetric analyzer (TGA), vibrating sample magnetometer (VSM), and Zeta potential analysis. The analytical parameters influencing the magnetic solid-phase fishing efficiency were optimized by univariate and multivariate methods (Box–Behnken design) by testing a positive control and celecoxib with active and inactive COX-2. Under the optimized ligand fishing conditions, twelve potential COX-2 inhibitors were screened and characterized in *Panax notoginseng* leaves. The results indicate that the proposed method provides a simple, feasible, selective, and effective platform for the efficient screening and identification of active compounds from Chinese herbal medicine. It has guiding significance for the synthesis and development of novel anti-inflammatory drugs, and provides a reference for the efficient discovery of anti-inflammatory drugs or lead compounds from the complex system of Chinese herbal medicine.

## 1. Introduction

Traditional Chinese medicine (TCM) plays an important role in the use of natural products to treat and prevent various diseases [1]. Many new drugs are derived from natural products and are widely used in clinical therapy. From natural products to innovative drugs, a critical step is the discovery and identification of bioactive lead compounds; however, bioactive molecules are unique, complex, and difficult to isolate, making their extraction from complex natural products challenging. The problem of how to screen active substances from natural products quickly and efficiently is a current research hotspot.

Traditional compound extraction and separation methods are heavy in workload and low in efficiency. With the development of bioinformation technology, some new effective component screening methods have emerged [2]. Ligand fishing technology based on receptor–ligand affinity was designed to attach targets such as enzymes and membrane proteins to the carrier material, and coupled with chromatography or mass spectrometry, has been recognized as a convenient and efficient way to rapidly screen active substances from natural products [3,4,5,6,7]. It is especially suitable for screening potential drugs’ active substances from multi-component systems and provides a new way for the development of new drugs, therefore, it has been widely considered [8].

The magnetic ligand fishing method uses magnetic nanoparticles (MNPs) modified with functional groups as the carrier, and the target protease is fixed on the MNPs by adsorption [9], embedding [10], cross-linking [11], or covalent bonding [12]. It is widely used because of its large specific surface area, good dispersion, high coupling capacity, super paramagnetism, and easy solid–liquid phase separation [13,14]. However, the problems of physical adsorption enzyme leakage, a decrease in covalent enzyme activity, and a low recovery rate of enzyme activity still need to be further improved [15].

Immobilized metal affinity magnetic nanotechnology is regarded as one of the most convenient methods for enzyme immobilization. It is mainly based on immobilized metal affinity chromatography (IMAC) technology and uses MNPs as a carrier, binding to protease by a coupling agent and chelating metal ions—such as Cu^2+^, Ni^2+^, Zn^2+^, and Co^2+^—and other transition metal ions to form immobilized metal affinity magnetic nanoparticles (IMAN) [16]. The direct loading of metal ions onto the surface of the carrier makes it exhibit a fairly high enzyme capacity and it retains most of the enzyme activity after fixation [17]. This technology not only has a higher binding efficiency and simple and fast operation but also can solve the above problems such as the decrease in protease activity after fixation.

During the preparation of IMAN, the use of coupling agents reduces the magnetic properties of IMAN and affects its ability to adsorb target enzymes. Moreover, some coupling agents are expensive and highly toxic [18]. It has been found that dopamine (DA) can be oxidized and polymerized on various solid surfaces, including polymers, metals, ceramics, etc., to form a polydopamine (PDA) film with strong hydrophilicity, good biocompatibility, good environmental stability, adjustable film thickness, and stable three-dimensional recognition sites. This makes it a material of choice for competitive new enzyme immobilization methods. The PDA layer contains rich phthalic groups, which are easily oxidized to a quinone structure under alkaline conditions and then react with molecules containing amino or sulfhydryl groups by Michael addition or Schiff base [19]. In addition, catechol groups can be complexed with metal ions, which facilitates the fixation of metal ions on the surface of MNPs [20,21]. Therefore, PDA, due to its excellent performance, is used to replace traditional coupling agents and chelating agents to modify MNPs and then combined with IMAN technology to achieve fixed-point fixation of COX-2, which integrates the advantages of MNPs, IMAN, and PDA to make up for their respective shortcomings. This will be a green and environmentally friendly anti-inflammatory fishing tool molecule with high enzyme activity, strong specificity, good stability, a strong magnetic response, and high sensitivity.

Inflammation is a very common and important pathological process that can cause many diseases [22]. The discovery of anti-inflammatory drugs and the treatment of inflammation are particularly important. Compared with traditional anti-inflammatory drugs, COX-2 inhibitors have better anti-inflammatory effects and significantly reduced adverse reactions [23]. COX-2 catalyzes the synthesis of prostaglandins from arachidonic acid [24]. It is an important target for the discovery and development of anti-inflammatory drugs. A large number of studies have shown that the effective ingredients in Chinese herbs, such as alkaloids, coumarins, flavonoids, and saponins, have good anti-inflammatory effects and have few toxic side effects [25]; however, how to quickly identify COX-2 inhibitors from traditional Chinese medicine is a key problem to be solved.

*Panax notoginseng* (Burk.) F. H. Chen is generally known as San qi in Chinese. Its roots can be used to treat trauma, body pain, inflammation, and cardiovascular diseases. At present, the demand for *Panax notoginseng* is rising year by year. However, after hundreds of years of cultivation, the adaptability of *Panax notoginseng* to the environment has gradually declined, and the problem of continuous cropping obstacles has become increasingly prominent. Moreover, the harvest of *Panax notoginseng* root requires long growth periods so the resources for *Panax notoginseng* are insufficient. Studies have shown that the *Panax notoginseng* leaves are rich in danmarane triterpenoid saponins, which indicates that *Panax notoginseng* leaves may be a substitute for the roots [26,27]. In order to expand the utilization rate of *Panax notoginseng* resources, the development and application of its leaves have gradually attracted the attention of researchers.

In this paper, the MNPs were modified by PDA, the coordination reaction between the PDA and metal ions was carried out, and then the fixed target enzyme COX-2 with metal affinity was used. After a series of characterization and performance investigations, the PDA-modified fixed COX-2 magnetic nano-fishing tool with excellent performance was prepared. The anti-inflammatory molecules in the total saponins of *Panax notoginseng* leaves were captured with this fishing tool. The captured anti-inflammatory components were separated and their structures were identified by modern chromatography and spectroscopy. The combined ligand fishing/HPLC-MS platform was used to identify potential COX-2 inhibitors in the crude extract of *Panax notoginseng* leaves. The site-directed immobilization strategy based on metal affinity bonds allows for controlling the enzyme orientation to avoid enzyme active site blocking, conformational changes, or denaturing during the immobilization.

## 2. Results and Discussion

### 2.1. Characterization of Fe_3_O_4_@C@PDA-Ni^2+^@COX-2 MNPs

**Figure 1:**
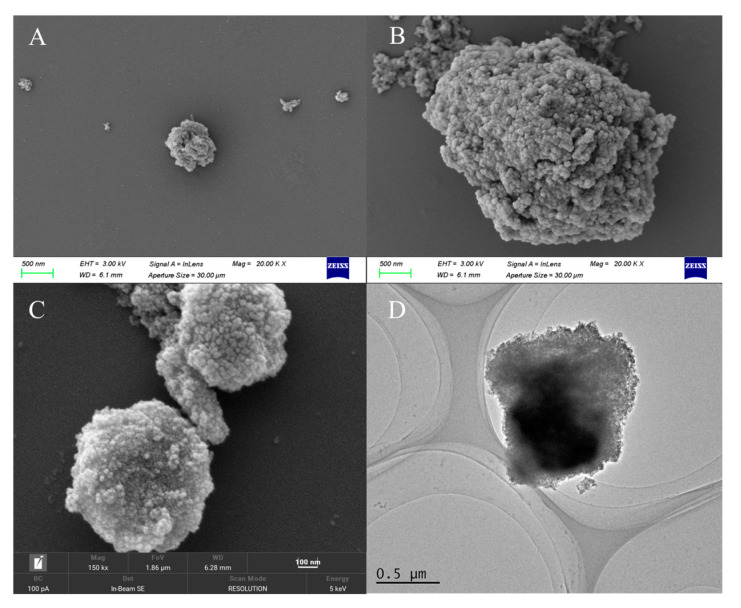
SEM image of (**A**) Fe_3_O_4_@C, (**B**) Fe_3_O_4_@C@PDA, and (**C**) Fe_3_O_4_@C@PDA-Ni^2+^; TEM image of (**D**) Fe_3_O_4_@C@PDA-Ni^2+^.

The morphology of the MNPs was characterized by SEM and TEM and the results are shown in Figure 1. The SEM characterization of Fe_3_O_4_@C (Figure 1A) shows that Fe_3_O_4_ is attached to the surface of carbon nanostructures with irregular shapes and multiple indentations, and the particle size is between 300 and 400 nm. After the coating of PDA, the particle size of the nanoparticles increases obviously and the surface has many pores. Fe_3_O_4_@C@PDA-Ni^2+^ is similar in appearance to Fe_3_O_4_@C@PDA, with a particle size of about 600 nm, and there are also many protrusions and microporous structures on the surface. The core-shell structure of the MNPs can be seen from the TEM image (Figure 1D), which indicates that PDA is successfully coated.

**Figure 2:**
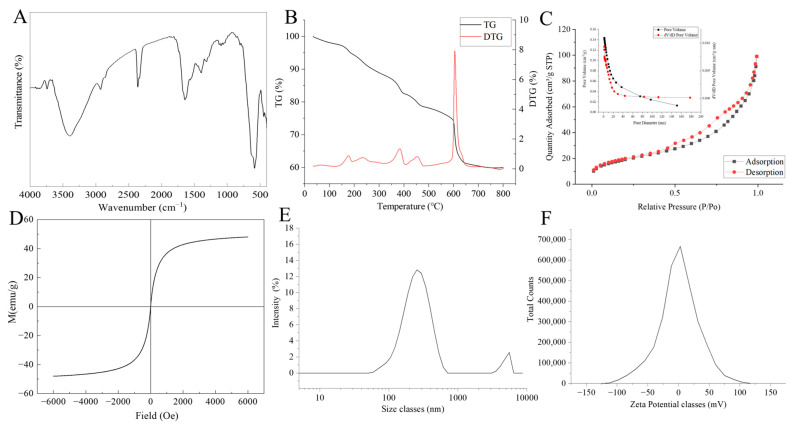
(**A**) FT-IR spectra; (**B**) TGA thermograms; (**C**) Nitrogen adsorption–desorption isotherms with pore diameter distribution (inset); (**D**) VSM magnetization curves; (**E**) particle size distribution curve; and (**F**) Zeta potential distribution diagram of Fe_3_O_4_@PDA-Ni^2+^@COX-2 NPs.

FT-IR is used to analyze the functional groups on the surface of the material. The results are shown in Figure 2A. The absorption peak between 3000 and 2800 cm^−1^ corresponds to the C-H bond in the alkane, and the absorption peak near 580 cm^−1^ is attributed to the tensile vibration of Fe-O in Fe_3_O_4_ [28]. The peaks at 1645 and 1397 cm^−1^ are attributed to the N-H deformation vibration on the surface of the PDA and the C=C resonance vibration in the aromatic ring, respectively, indicating the successful coating of the PDA [29,30].

It can be seen from the TG-DTG curve of the MNPs (Figure 2B) that the quality of the sample is gradually lost with the increase in temperature. From room temperature to 200 °C, the sample mass has a small loss; it is speculated that the sample mass decline from room temperature to 200 °C is mainly caused by the evaporation of water in the sample, and at 200–400 °C and 400–500 °C, these two stages may be the Ni^2+^ and PDA layer decomposition leading to sample mass loss. At the stage of 500–650 °C, the mass of the sample first slowly decreases and then sharply decreases. It is inferred that with the increase in temperature, the Carbon layer coated on the Fe_3_O_4_ is completely decomposed at about 600 °C, leaving Fe_3_O_4_ with no significant change in mass. The thermal gravimetric analysis results show that the MNPs displayed excellent thermal stability below 200 °C. Additionally, it can be seen that the carbon layer and PDA layer were successfully coated, and Ni^2+^ was successfully chelated with PDA.

The surface area and porosity of MNPs were investigated via Brunauer–Emmet–Teller (BET) analysis. The nitrogen adsorption–desorption isotherm of the sample is a typical type II isotherm (Figure 2C). At the low pressure of 0~0.2 P/P0, the absorption volume of the sample is relatively low, indicating that the mesopore is dominant in the nano-composites. The calculated surface area of the nano-composite is 69.736 m^2^/g. Results of the pore size distribution show that the pore size distribution of the sample is centered at 8.79 nm, confirming the presence of mesoporous structures in the prepared nano-composites.

The magnetic properties of MNPs were analyzed by VSM analysis (Figure 2D), which is a typical S-shaped M-H curve. The saturation magnetization (Ms) is 48.01 emu/g, indicating that the nanomaterials still have strong magnetic properties after being coated with carbon and PDA layers.

According to the analysis results of particle size distribution (Figure 2E), it can be seen that the particle size distribution of the sample is uniform, with an average particle size of 255 nm. In addition, the particle size distribution diagram shows a peak at 5559 nm, which may be caused by the gap between stacked particles. According to the Zeta potential distribution diagram (Figure 2F), it can be seen that the Zeta potential of the sample is 0.359 mV, and the Zeta potential distribution of the sample is relatively concentrated, indicating that the prepared sample has good stability.

From the XPS diagram of the MNPs (Figure S1), the element composition, valence state, and proportion of the magnetic nanospheres can be seen. The peaks corresponding to C 1*s*, N 1*s*, O 1*s*, Ni 2*p*, and Fe 2*p* can be seen in the XPS wide-spectrum scan graph (Figure S1A) as well as the narrow-spectrum scan of each element (Figure S1B–F), proving the existence of Fe_3_O_4_, PDA, and Ni^2+^ in the synthesized material. In addition, the atomic ratio of each element is calculated (Table S1, Supplementary Materials).

### 2.2. Validation of the Ligand Fishing Assay

The ligand fishing experiment was developed and optimized using an equimolar mixture of celecoxib, indomethacin, and glipizide. Indomethacin, a non-specific COX-2 inhibitor, and celecoxib, which is a well-known specific high-affinity inhibitor of COX-2, was used as positive controls. Additionally, glipizide, which does not interact with COX-2, was used as a negative control to verify the selectivity of the prepared fishing tools. The mixture of celecoxib, indomethacin, and glipizide (Figure S2A), the ligand fished by Fe_3_O_4_@C@PDA-Ni^2+^@COX-2 (Figure S2B), and the supernatant after ligand fishing (Figure S2C) were obtained and analyzed by HPLC. The results are shown in Figure S2 of the Supplementary Materials, which revealed that glipizide and indomethacin almost did not bind to the COX-2 enzyme as they were present in the residual solution after mixed fishing. On the contrary, celecoxib was clearly observed in the immobilized COX-2 elution solution, which showed that celecoxib was successfully maintained and fished out with the highest amount and confirmed the specificity of the proposed method.

### 2.3. COX-2 Activity Assay for the Immobilized Enzyme

**Figure 3:**
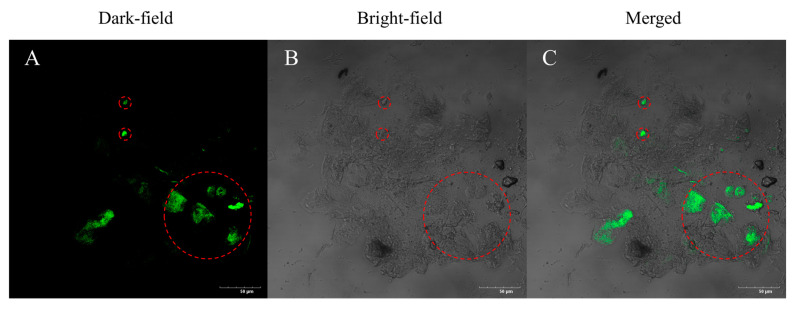
Confocal laser scanning images of immobilized COX-2 using MNPs in (**A**) bright-field, (**B**) dark-field, and (**C**) merged bright-darkfield. The bar is 50 μm.

To determine whether COX-2 was immobilized by the MNPs, the samples were stained with Fluorescein Isothiocyanate (FITC) and imaged with a confocal laser scanning microscope (CLSM), as shown in Figure 3. The green fluorescence emitted by stained COX-2 in the MNPs can be obviously observedwithin the red dashed circles in the Figure 3, indicating that COX-2 is successfully fixed on the MNPs.

### 2.4. Optimization of Conditions of Magnetic Solid-Phase Fishing (MSPF)

The recovery of celecoxib was used to evaluate the capability for extraction with the Fe_3_O_4_@C@PDA-Ni^2+^@COX-2 MNPs. In order to achieve the best extraction efficiency, a single-factor variable method and Box–Behnken design (BBD) were adopted. Several experimental parameters involving the amount of Fe_3_O_4_@C@PDA-Ni^2+^@COX-2, the volume of COX-2, extraction time, extraction temperature, desorption solvent, and desorption time were studied in detail. All experiments were performed in triplicate. The extraction recovery was calculated based on the following equations:

$$\mathrm{Recovery}\left(\%\right)=\frac{C_{a}\;\text{×}\;V_{a}}{C_{0}\;\text{×}\;V_{0}}\;\text{×}\;100$$

where *C*_a_ and *C*_0_ are the concentration of analyte in the extraction phase and the initial analyte concentration in the sample solution, respectively; *V*_a_ and *V*_0_ are the volumes of the extraction phase and sample solution, respectively.

#### 2.4.1. Effect of the Concentration of Ni^2+^

**Figure 4:**
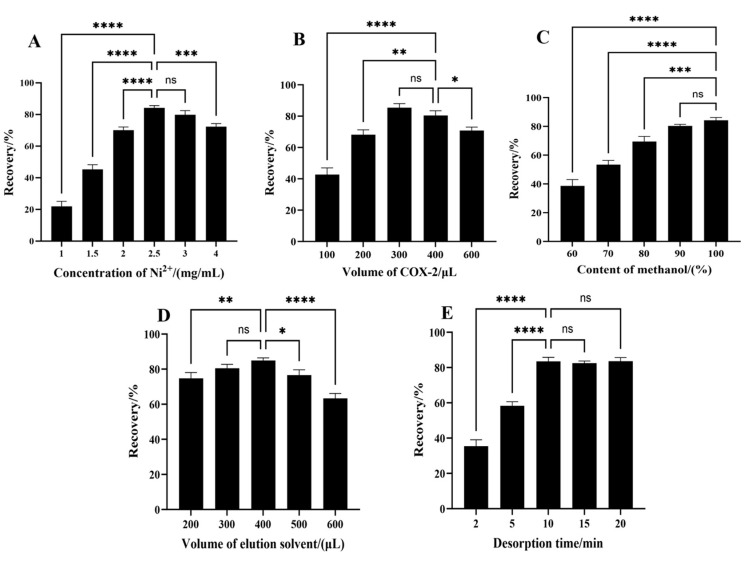
Effects of (**A**) concentration of Ni^2+^, (**B**) volume of COX-2, (**C**) content of methanol, (**D**) volume of elution solvent, and (**E**) desorption time. The data shown are the mean ± SD, *n* = 3. One-way analysis of variance (ANOVA) was used, *p* < 0.05 was considered as statistically significant (**** *p* < 0.0001, *** *p* < 0.001,** *p* < 0.01, * *p* < 0.05, ns *p* > 0.05).

The concentration of Ni^2+^ was estimated for the synthesis of Fe_3_O_4_@C@PDA-Ni^2+^@COX-2 MNPs to improve their fishing capability. The effects of different Ni^2+^ concentrations—including 1, 1.5, 2, 2.5, 3, and 4 mg/mL—on the fishing effect of ligands were investigated, and the results are shown in Figure 4A. With the increase in Ni^2+^ concentration, the recovery gradually increased, which was highest at the concentration of 2.5 mg/mL, and when the concentration exceeded 2.5 mg/mL, the recovery decreased sharply. It was speculated that the excessive concentration of Ni^2+^ would destroy the PDA layer on the surface of MNPs, resulting in a decrease in the amount of enzyme fixation. Therefore, 2.5 mg/mL of Ni^2+^ was selected to synthesize Fe_3_O_4_@C@PDA-Ni^2+^@COX-2 MNPs.

#### 2.4.2. Effect of the Volume of COX-2

The volume of COX-2 added during the preparation of MNPs also affects the fishing efficiency. The effect of the volume of COX-2 ranging from 100 to 600 μL was investigated. The results are shown in Figure 4B. It can be seen that the extraction efficiency increased with the increase in volume of COX-2 from 100 to 300 μL but decreased with further increases in volume of COX-2. The initial increase is due to the addition of more reactant, which promotes binding and increases the amount of immobilized enzyme on the material; however, too many enzymes in the solution are not good for enzyme fixation. Therefore, the optimized volume of COX-2 was 300 μL.

#### 2.4.3. Effect of the Type of Elution Solvent

In order to obtain better recoveries, different proportions of methanol as the elution solvent were optimized. As shown in Figure 4C, with the increase in methanol proportion, the elution amount of celecoxib gradually increased and the elution efficiency showed an obvious upward trend. This indicates that a high proportion of methanol, especially 100% methanol, was more conducive to breaking the hydrogen bond between COX-2 and the target substance, promoting the dissociation of the target substance and improving the fishing efficiency. Therefore, 100% methanol was selected as the optimal elution solvent.

#### 2.4.4. Effect of the Volume of Elution Solvent

The volume of the elution solvent was studied in the range of 200 to 600 μL, and the experimental results are shown in Figure 4D. It can be seen that the elution efficiency is positively correlated with the amount of eluent, and reaches its highest at 400 μL, indicating that higher methanol volume can provide more hydroxyl groups to form new hydrogen bonds with proteins and break existing hydrogen bonds, promoting more celecoxib and COX-2 dissociation, although the elution efficiency decreases when the volume of eluent continues to increase [31]. It is speculated that the eluent of 400 μL has basically eluded the target substance completely, so when the volume increases, the concentration of the target substance in the eluent decreases; therefore, the volume of the final elution solvent is determined to be 400 μL.

#### 2.4.5. Effect of the Desorption Time

The influence of desorption time was also studied in the range of 2–20 min. The highest extraction efficiency was achieved at 10 min, and there were no significant changes with longer desorption times (Figure 4E). Therefore, 10 min was implemented in subsequent experiments.

#### 2.4.6. Experimental Design

**Figure 5:**
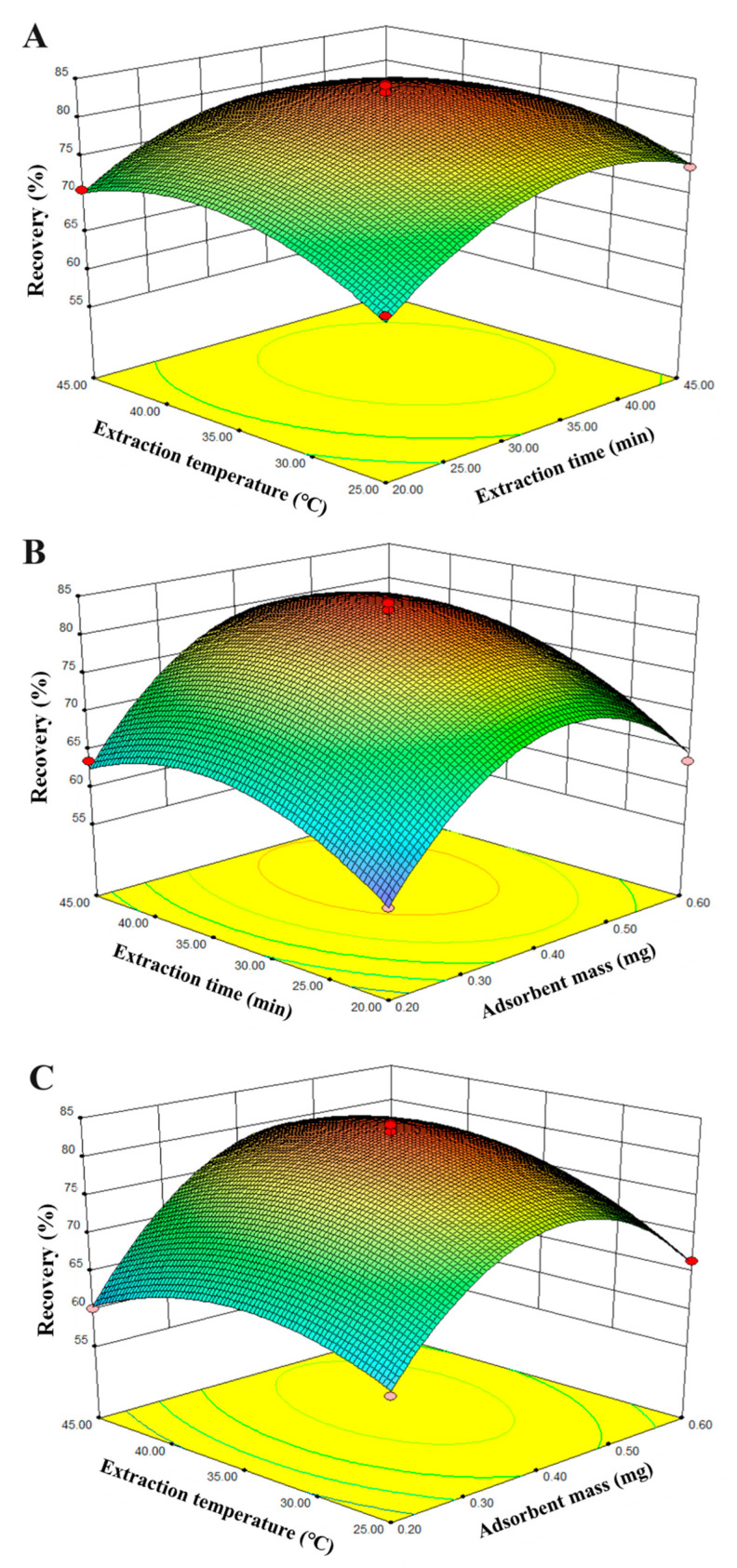
Three-dimensional response surface variables for extraction of celecoxib: (**A**) extraction time and extraction temperature; (**B**) extraction time and adsorbent mass; and (**C**) extraction temperature and adsorbent mass.

The results of a 17-run BBD experimental design are shown in Table 1 and Figure 5, displaying the effects of different parameters on the extraction yield of celecoxib. With the celecoxib extraction recovery as the independent variable, the mathematical model obtained is outlined below:

Y = 82.16 + 4.38X_1_ + 3.33X_2_ + 1.48X_3_ − 0.37X_1_X_2_ + 1.98X_1_X_3_ − 1.28X_2_X_3_ − 11.92X_1_^2^ − 6.27X_2_^2^ − 4.97X_3_^2^

The analysis of variance (ANOVA) of the regression model demonstrates that the model is highly significant. The value of R^2^ = 0.9883 indicates a good correlation between the experimental and predicted values of the recoveries. The *p* value, F value, and lack of fit value of the model is lower than 0.0001 (significant), 35.02, and 0.8384 (not significant), respectively. These values confirm that the model fitness is good and the quadratic model is statistically significant for the response.

From Figure 5 and the data, it can be concluded that the number of MNPs and extraction time presented remarkable effects on the celecoxib recoveries. The extraction recoveries of celecoxib increase with the increase in the extraction time and the number of MNPs, and then, no obvious changes are observed. Based on the BBD experiments, the optimal conditions are as follows: 0.45 mg of the amount of adsorbent, 35 min of extraction time, and 36 °C of extraction temperature. The optimal results obtained by experiments are consistent with the predicted values, which verified the feasibility of the method.

### 2.5. Adsorption Kinetics

In order to investigate the adsorption properties of the prepared fishing tool, the data of the celecoxib adsorption kinetics experiment with magnetic immobilized COX-2 were linearly fitted with the pseudo-first-order kinetic model and the pseudo-second-order kinetic model to establish the corresponding kinetic models.

The pseudo-first-order kinetics is based on the condition that the adsorption process is mainly physical adsorption, and the formula of the model is as follows.

(1) $$l\mathrm{og}(q_{e}-q_{t})=l\mathrm{og}q_{e}-\frac{k_{1}}{2.303}t$$

The pseudo-second-order kinetic equation assumes that the adsorption type is chemisorption, and the formula of the model is the following:

(2) $$\frac{t}{q_{t}}=\frac{1}{k_{2}q_{e}^{2}}+\frac{t}{q_{e}}$$

where q_t_ is the adsorption capacity of the adsorbent to celecoxib at time t, mg/g; q_e_ is the equilibrium adsorption capacity of celecoxib adsorbent, mg/g; t is the time of celecoxib adsorption by the adsorbent, min; k_1_ is the adsorption rate constant of the pseudo-first-order kinetics, min^−1^; k_2_ is the adsorption rate constant of the pseudo-second-order kinetics, g/(mg·min).

**Figure 6:**
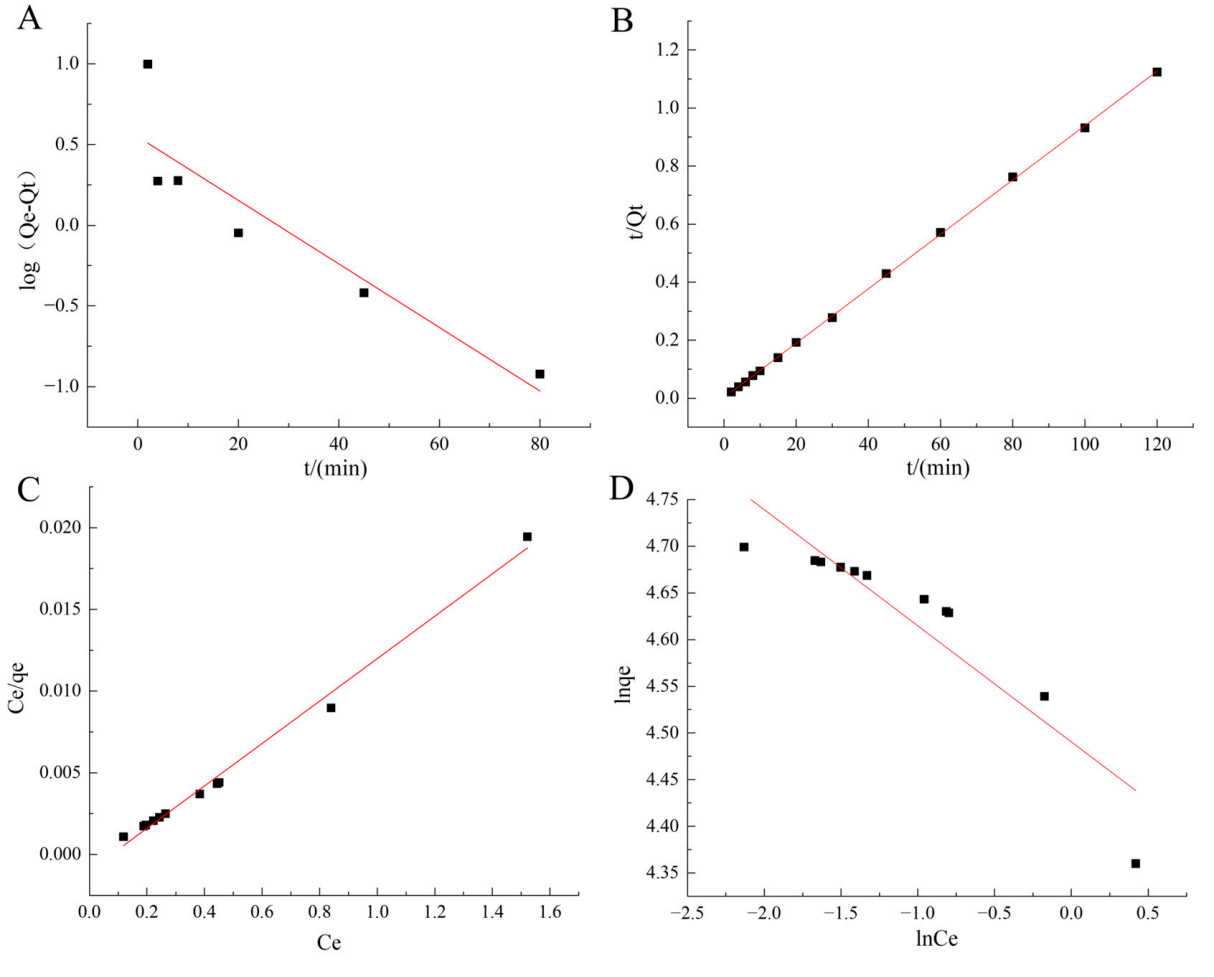
(**A**) The pseudo-first-order kinetic model and (**B**) the pseudo-second-order kinetic model fitting diagram; (**C**) the Langmuir isothermal adsorption model; and (**D**) Freundlich isothermal adsorption model fitting diagram.

The fitting curve of the obtained pseudo-first-order model (Figure 6A) and pseudo-second-order model (Figure 6B) is shown in Figure 6, and the corresponding fitting parameters are shown in Table S2 of the Supplementary Materials. It can be seen from Table S2 and Figure 6 that the R^2^ value of the pseudo-first-order kinetic fitting curve is 0.7988 and the R^2^ value of the pseudo-second-order kinetic fitting curve is 0.9998, which shows that the second-order kinetic fitting results are better and that the equilibrium adsorption capacity calculated by the pseudo-second-order kinetic model is closer to the experimental results. Therefore, the adsorption of celecoxib by immobilized COX-2 is more consistent with the pseudo-second-order kinetic model, that is, chemisorption is the main method.

### 2.6. Adsorption Equilibrium

The Langmuir isothermal adsorption model and Freundlich isothermal adsorption model were used to investigate the celecoxib adsorption equilibrium behavior at room temperature [32].

The assumed condition of the Langmuir isotherm is adsorption on the surface of the monolayer. The equation is as follows.

(3) $$\frac{C_{e}}{q_{e}}=\frac{C_{e}}{q_{m}}+\frac{1}{q_{m}k_{L}}$$

The Freundlich isothermal adsorption model can be used for both single-molecular layer adsorption and multi-molecular layer adsorption, and the equation is as follows.

(4) $$\ln q_{e}=\ln K_{F}+\frac{1}{n}\ln C_{e}$$

where C_e_ is the concentration when the adsorption of celecoxib reaches equilibrium, mg/L; q_e_ is the amount of celecoxib adsorbed by magnetically immobilized COX-2, mg/g; q_max_ is the adsorption amount when the adsorption reaches saturation, mg/g; k_L_ is the Langmuir constant, L/mg; K_F_ and n are related to the adsorption capacity and intensity, respectively.

The data were fitted into two isothermal adsorption models. The fitting results are shown in Figure 6C,D, and the calculated fitting parameters are shown in Table S3. It can be seen that the R^2^ value of the Langmuir isothermal adsorption model (0.991) is higher than that of the Freundlich isothermal adsorption model (0.8325), and the saturation adsorption capacity fitted by the Langmuir isothermal adsorption model is closer to the experimental results, indicating that the adsorption process of immobilized COX-2 on celecoxib is monolayer adsorption.

### 2.7. Method Validation

#### 2.7.1. Limits of Detection and Quantification

Celecoxib standard solution was used to obtain the standard curves. The concentration (c, mg/mL) of celecoxib is the horizontal coordinate X and the measured peak area (A) is the vertical coordinate Y. The linear regression equation was A = 7.711 × 10^6^C + 7.100 × 10^5^ with a correlation coefficient of 0.9996. The limits of detection (LODs) and quantification (LOQs) are determined on the basis of a signal-to-noise (S/N) ratio of 3 and 10, respectively. The LODs and LOQs of celecoxib were 0.037 and 0.124 mg/mL.

#### 2.7.2. Precision and Recovery

The intra-day precision was obtained by determining the analytes five times in day, and the inter-day precision was obtained by performing the same process in five consecutive days. The intra-day RSDs and inter-day RSDs are 3.3 and 2.9, which indicate the method possessed excellent precision.

### 2.8. Ligand Fishing and LC-MS of Ligands

The UPLC-Q-TOF-MS chromatograms of the original extract of *Panax notoginseng* leaves (A) and the elution fractions after ligand fishing by COX-2 (B) were compared (Figure S3, Supplementary Materials). The UPLC-Q-TOF-MS chromatogram of eluent fractions after COX-2 ligand fishing was significantly simpler and clearer than that of the unfished extracts, implying that considerable substances without affinities to COX-2 were washed off and COX-2 MNPs showed efficient separation of the target components.

After fishing, 12 compounds were obtained and identified as ligand compounds from the extract of *Panax notoginseng* leaves. Their retention times and molecular ion peaks are listed in Table 2. The structures of the 12 compounds were elucidated by comparison with previously reported MS data and shown in Figure S4 of the Supplementary Materials.

## 3. Materials and Methods

### 3.1. Chemicals and Reagents

Ferric nitrate (Fe(NO_3_)_3_·9H_2_O) (98.5%) and poly (vinyl alcohol) were supplied from Chengdu Chemical Factory (Chengdu, China). Dopamine hydrochloride was purchased from Aladdin Chemicals (Shanghai, China). Ethylene glycol (AR) was obtained from Fuyu Fine Chemical Company (Tianjin, China). Analytical reagent-grade Nickel Chloride was obtained from Beijing Chemicals (Beijing, China). Celecoxib, Glipizide, and indomethacin (HPLC ≥ 98%) were purchased from Beijing Solaibao Technology Co., Ltd. (Beijing, China). Fluorescein Isothiocyanate (FITC) was purchased from Shanghai Maclin Biochemical Technology Co., Ltd. (Shanghai, China). Human recombinant cyclooxygenase-2 (COX-2) was purchased from Wuhan Aibotek Biotechnology Co., Ltd. (Wuhan, China).

### 3.2. Instrumentation

Teflon-lined stainless steel autoclave (Xi’an Changyi Instrument Equipment Co., Ltd., Xi’an, China), Fourier-transform infrared spectroscopy (FT-IR, Cary 630, Agilent Technologies Inc., Palo Alto, CA, USA), scanning electron microscopy (SEM, Hitachi SU8000, Tokyo, Japan), transmission electron microscopy (TEM, TF 20, Tokyo, Japan), X-ray photoelectron spectroscopy (XPS, Thermo Scientific, ESCALAB 250Xi, Carlsbad, CA, USA), and a vibrating sample magnetometer (VSM, Meghnatis Kavir Kashan Co., Kashan, Iran) were used to characterize the NPs. A confocal laser scanning microscope (CLSM, Olympus FV3000, Tokyo, Japan) was used to photograph the dyed Fe_3_O_4_@C@PDA-Ni^2+^@COX-2. An HPLC instrument (Agilent Technologies Inc., Palo Alto, CA, USA) equipped with UV detector was used for quantitative analysis. Additionally, an ultra-high-performance liquid-phase quadrupole time-of-flight mass spectrometer was employed (Waters, Milford, MA, USA, Xevo G2-XS QTof).

### 3.3. Samples Treatment

The dried *Panax notoginseng* leaves were extracted 3 times by reflux heating with 50% ethyl alcohol (EtOH) solution and the filtrate was concentrated after consolidation. The concentrated extract was adsorbed by D101 macroporous resin for 12 h, and then eluted with distilled water, 30% EtOH, and 80% EtOH in turn. The 80% EtOH eluent was collected and concentrated to obtain the total saponin extract from the *Panax Notoginseng* leaves.

### 3.4. Preparation of Magnetic Fishing Tool

**Figure 7:**
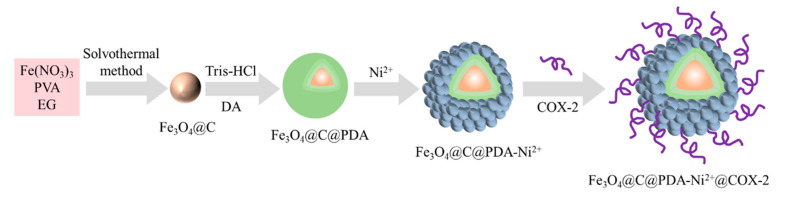
Schematic of preparation of Fe_3_O_4_@C@PDA-Ni^2+^@COX-2.

The process for the preparation of Fe_3_O_4_@C@PDA-Ni^2+^@COX-2 is shown in Figure 7.

#### 3.4.1. Synthesis of Fe_3_O_4_@C

Fe_3_O_4_@C NPs were prepared by a one-step hydrothermal reaction [33]. Briefly, solution A (4.58 g of Fe(NO_3_)_3_·9H_2_O was dissolved in 20 mL of water) was slowly added into solution B (4.00 g of polyvinyl alcohol (PVA) was dissolved in 20 mL of water). After being vigorously stirred, 120 mL of ethylene glycol was added to the above solution. Then, the mixture was directly added into a Teflon-lined stainless steel autoclave at 200 °C for 12 h. The product was separated by a strong magnet, purified with water and ethanol 3 times, and then dried in vacuum freeze-drying equipment. Then, the Fe_3_O_4_@C NPs were obtained.

#### 3.4.2. Synthesis of Fe_3_O_4_@C@PDA

A total of 400 mg of Fe_3_O_4_@C NPs and 800 mg of DA·HCl (2 mg/mL, pH 8.5) were dispersed into 400 mL of water. The solution was agitated with a vortex mixer at room temperature for 12 h. After washing and drying, Fe_3_O_4_@C@PDA NPs were obtained.

#### 3.4.3. Metal Ion Affinity-Oriented Immobilization

A total of 200 mg of the dried Fe_3_O_4_@C@PDA was dispersed in 200 mL NiCl_2_ solution (2.5 mg/mL), stirred by magnetic force at room temperature for 12 h, washed with ultra-pure water several times after magnetic separation until the clear liquid was colorless and clarified, then dried at 50 °C in a vacuum drying oven to obtain Fe_3_O_4_@C@PDA-Ni^2+^ composite NPs.

#### 3.4.4. Immobilization of COX-2

A total of 0.4 mg of Fe_3_O_4_@C@PDA-Ni^2+^ was weighed and balanced in Tris-HCl buffer (10 mM, pH 8.0) for 1 h, then added to 500 μL of Tris-HCl buffer containing COX-2 (0.08 μg), and incubated at 4 °C for 2 h. The Fe_3_O_4_@C@PDA-Ni^2+^ fixed to the COX-2 was collected by magnetic separation, washed with ultra-pure water to remove unreacted components, and stored in Tris-HCl buffer at 4 °C for further use.

### 3.5. Establishment and Validation of Ligand Fishing Assay

**Figure 8:**
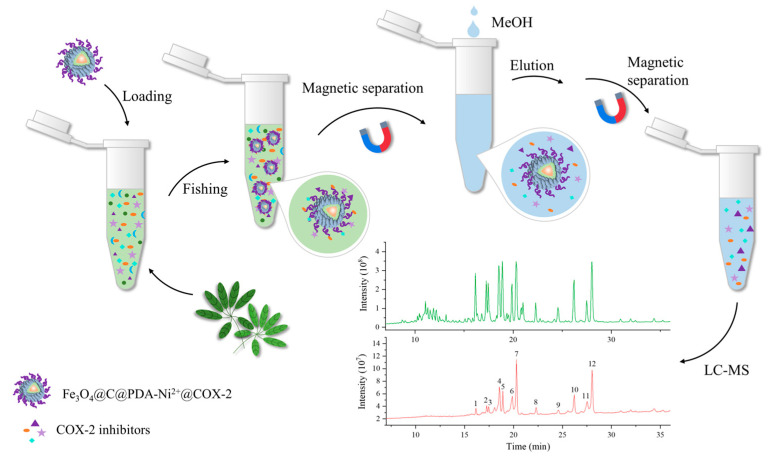
MSPF procedure. Peaks 1–12 are fishing compounds that are potential COX-2 inhibitors.

A schematic diagram of the ligand fishing procedure is illustrated in Figure 8. Firstly, loading occurred—a number of nanoparticles and Tris buffer were added to the samples. Subsequently, fishing—the mixture was incubated with continuous shaking. After 35 min at 36 °C, the supernatant was removed after magnetic separation of the MNPs. Finally, elution—the MNPs were washed three times with buffer solution. In order to elute the bound ligands from the immobilized enzyme, the MNPs were incubated with 400 μL of methanol (MeOH) as the elution solvent. After separation, the supernatant was filtered through a membrane (0.45 mm, PTFE) for HPLC analysis.

The column temperature was set at 25 °C. The eluent flow rate was 1.0 mL/min. The mobile phase consisted of solvent A (methanol) and solvent B (0.1%, *v*/*v*, formic acid/water). The chromatographic elution condition was 85% A at 0–6 min. The sample injection volume was 10.0 μL. The detection wavelength was 254 nm.

### 3.6. Application of Ligand Fishing in Crude Extract

The MNPs (4 mg/mL) were added into the above crude extract sample and Tris buffer solution (10 mM, pH 8.0). The mixture was cultured for 35 min under continuous oscillation at 36 °C. Magnetically separated MNPs were collected and washed three times with buffer solution. Then, they were incubated in 400 μL of methanol for 10 min to eluate the specific binding compounds. Finally, the eluent was analyzed by UPLC-Q-TOF-MS for the composition and structure of the compounds.

### 3.7. Analysis of Ligands by LC-MS

LC-MS technology was used to analyze the obtained ligands. The chromatographic conditions were as follows: The chromatographic column was C18 (100 mm × 2.1 mm, 1.7 μm). Mobile phases A and B were acetonitrile and 0.1% formic acid aqueous solution, respectively. The gradient conditions were as follows—0–5 min, 20–25% A; 5–20 min, 25–40% A; 20–25 min, 40–70% A; 25–27 min, 70–100% A; 27–35 min, 100% A; 35–37 min, 100–20% A; 37–45 min, 20% A. The detection wavelength was 254 nm, the injection volume was 5 μL, the flow rate was 0.3 mL/min, and the column temperature was 35 °C. Mass spectrum conditions: The ion source was the ESI ion source, using a negative ion mode detection, the quality scanning range was *m*/*z* 300–1400; capillary voltage: 3000 V; hole and cone voltage: 30 V; ion source temperature: 120 °C; desolvent temperature: 350 °C; atomizing gas flow rate: 50 L/h; desolvent gas (N_2_) flow rate: 500 L/h; collection time: 0–45 min; scanning time: 1.0 s.

### 3.8. Box–Behnken Design

In order to evaluate the interaction between adsorption conditions, the Box–Behnken design (BBD) with a three-level incomplete factorial design was used [34,35]. The adsorbent mass (X_1_), extraction time (X_2_), and extraction temperature (X_3_) are the independent variables, and the actual numerical values are shown in Table 1. All the acquired results were used for the computer simulation programming, applying the quadratic (second-degree) polynomial equation. For the three independent variables, the equation is outlined below:

Y = β_0_ + β_1_X_1_ + β_2_X_2_ + β_3_X_3_ + β_12_X_1_X_2_ + β_13_X_1_X_3_+ β_23_X_2_X_3_ + β_11_X_1_^2^ + β_22_X_2_^2^ + β_33_X_3_^2^

## 4. Conclusions

In this paper, a new strategy based on an Fe_3_O_4_@C@PDA-Ni^2+^@COX-2 ligand fishing combination with UPLC-Q-TOF-MS analysis was established to rapidly screen and characterize COX-2 inhibitors from natural products. Ni^2+^ affinity-oriented immobilization conditions are mild enough to maintain enzyme activity and spatial conformation. The proposed method was validated with celecoxib, a COX-2 selective inhibitor, which showed the method could successfully fish out COX-2 ligands. Under the optimal conditions by the MSPF method, twelve ligands of COX-2 inhibitors were rapidly extracted and identified from *Panax notoginseng* leaves. This method is more effective, simpler, and cheaper. In the future, the present ligand fishing approach can be applied to screen more bioactive components from complex natural products.

## Figures and Tables

**Table 1:** Experimental results based on BBD.

Experiments	Coded Levels			Response: Fishing Efficiency (%)
	X1 Amount of Adsorbent (mg)	X2 Extraction Time (min)	X3 Extraction Temperature (°C)	Celecoxib
1	0.40	32.50	35.00	79.8
2	0.40	32.50	35.00	79.4
3	0.60	45.00	35.00	72.1
4	0.20	32.50	45.00	60.1
5	0.20	20.00	35.00	56.6
6	0.40	32.50	35.00	84.1
7	0.40	20.00	45.00	70.7
8	0.40	32.50	35.00	83.2
9	0.40	32.50	35.00	84.3
10	0.40	20.00	25.00	65.6
11	0.20	45.00	35.00	63.6
12	0.40	45.00	25.00	73.7
13	0.20	32.50	25.00	60.7
14	0.40	45.00	45.00	73.7
15	0.60	20.00	35.00	63.6
16	0.60	32.50	25.00	66.5
17	0.60	32.50	45.00	73.8

**Table 2:** UPLC-Q-TOF-MS data of ligands found in fungal extracts.

No.	t/min	Mass [M − H]^−^ (*m*/*z*)	Error (ppm)	Formula	Compounds	Fragment Ions (*m*/*z*)
1	16.16	1239.6445	5.7	C_59_H_100_O_27_	Notoginsenoside Fa	1107.5992 [M-H-Xyl]^−^, 945.5453 [M-H-Xyl-Glc]^−^, 783.4907 [M-H-Xyl-2Glc]^−^, 621.4360 [M-H-Xyl-3Glc]^−^, 459.3853 [M-H-Xyl-4Glc]^−^,
2	17.26	1209.6321	4.4	C_58_H_98_O_26_	Notoginsenoside FP_2_	1077.5894 [M-H-Xyl]^−^, 945.5441 [M-H-Xyl-Ara]^−^, 783.4907 [M-H-Xyl-Ara-Glc]^−^, 621.4371 [M-H-Xyl-Ara-2Glc]^−^, 459.3871 [M-H-Xyl-Ara-3Glc]^−^,
3	17.45	1107.5994	3.9	C_54_H_92_O_23_	Ginsenoside Rb_1_	945.5443 [M-H-Glc]^−^, 783.4905 [M-H-2Glc]^−^, 621.4364 [M-H-3Glc]^−^, 459.3828 [M-H-4Glc]^−^,
4	18.55	1077.5879	3.2	C_53_H_90_O_22_	Ginsenoside Rc	945.5449 [M-H-Ara]^−^, 783.4908 [M-H-Ara-Glc]^−^, 621.4373 [M-H-Ara-2Glc]^−^, 459.3831 [M-H-Ara-3Glc]^−^,
5	18.91	1209.6311	3.6	C_58_H_98_O_26_	Notoginsenoside Fc	1077.5874 [M-H-Xyl]^−^, 945.5399 [M-H-2Xyl]^−^, 783.4904 [M-H-2Xyl-Glc]^−^, 621.4391 [M-H-2Xyl-2Glc]^−^, 459.3851 [M-H-2Xyl-3Glc]^−^,
6	19.89	1077.5873	2.6	C_53_H_90_O_22_	Ginsenoside Rb_2_	945.5439 [M-H-Ara]^-^, 783.4896 [M-H-Ara-Glc]^−^, 621.4368 [M-H-Ara-2Glc]^−^, 459.3907 [M-H-Ara-3Glc]^−^,
7	20.30	1077.5876	2.9	C_53_H_90_O_22_	Ginsenoside Rb_3_	945.5433 [M-H-Xyl]^−^, 783.4902 [M-H-Xyl-Glc]^−^, 621.4361 [M-H-Xyl-2Glc]^−^, 459.3870 [M-H-Xyl-3Glc]^−^,
8	22.29	945.5463	4.2	C_48_H_82_O_18_	Ginsenoside Rd	783.4873 [M-H-Glc]^−^, 621.4349 [M-H-2Glc]^−^, 459.3892 [M-H-3Glc]^−^
9	24.56	945.5447	2.5	C_48_H_82_O_18_	Gypenoside XVII	783.4891 [M-H-Glc]^−^, 621.4352 [M-H-2Glc]^−^, 459.3838 [M-H-3Glc]^−^
10	26.21	915.5348	3.4	C_47_H_80_O_17_	Notoginsenoside Fe	783.4901 [M-H-Ara]^−^, 621.4360 [M-H-Ara-Glc]^−^, 459.3873 [M-H-Ara-2Glc]^−^
11	27.48	915.5337	2.2	C_47_H_80_O_17_	Vina-ginsenoside R_18_	783.4898 [M-H-Xyl]^−^, 621.4356 [M-H-Xyl-Glc]^−^, 459.3799 [M-H-Xyl-2Glc]^−^
12	28.02	915.5372	6.0	C_47_H_80_O_17_	Gypenoside IX	783.4895 [M-H-Xyl]^−^, 621.4373 [M-H-Xyl-Glc]^−^, 459.3802 [M-H-Xyl-2Glc]^−^

## Data Availability

The data are contained within the article and the Supplementary Materials.

## Supplementary Materials

The following supporting information can be downloaded at: https://www.mdpi.com/article/10.3390/molecules29235801/s1, Figure S1: XPS images of Fe_3_O_4_@C@PDA-Ni^2+^@COX-2, (A) XPS wide spectrum scan graph, (B–F) XPS narrow spectrum scan graph; Figure S2: HPLC Chromatogram of (A) mixed solution consisting of glipizide (1), indomethacin (2), and celecoxib (3), (B) ligand fished by Fe_3_O_4_@C@PDA-Ni^2+^@COX-2 and (C) residual solution after mixed fishing; Figure S3: Total ion chromatograms of (A) *Panax quinquefolium* extract and (B) fishing liquid; Figure S4: Structures of compounds 1–12; Table S1: The atomic ratios of XPS analysis of Fe_3_O_4_@C@PDA-Ni^2+^@COX-2; Table S2: Parameters of the pseudo-first-order kinetic model and pseudo-second-order kinetic model; Table S3: Parameters of the Langmuir isothermal adsorption model and Freundlich isothermal adsorption model.
