# Supplementary material for: A Novel Platform Featuring Nanomagnetic Ligand Fishing Based on Fixed-Orientation Immobilized Magnetic Beads for Screening Potential Cyclooxygenase-2 Inhibitors from Panax notoginseng Leaves
Source: Molecules. 2024 Dec 9;29(23):5801. doi: 10.3390/molecules29235801 (PMC11643511; doi:10.3390/molecules29235801)
Supplement: Supplementary file 1 [file molecules-29-05801-s001.zip › molecules-3232263-supplementary.pdf]

Figure S1 XPS images of Fe_3O_4@C@PDA-Ni^{2+}@COX-2, (A)XPS wide spectrum scan graph, (B-F)XPS narrow spectrum scan graph.

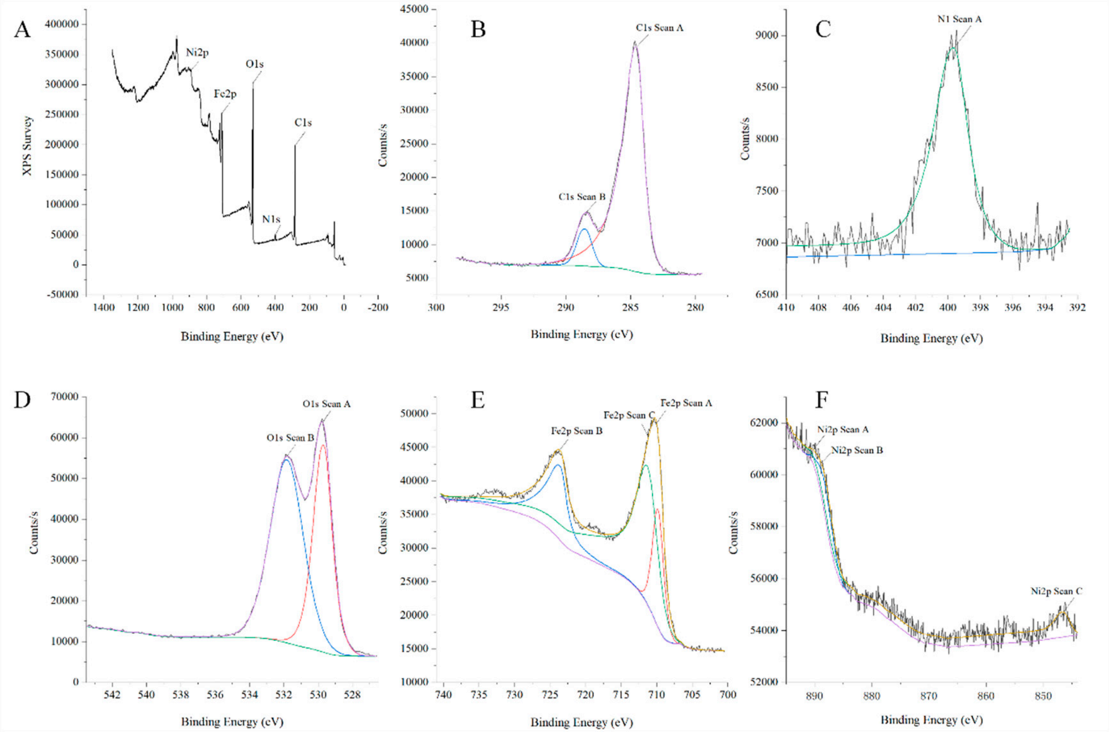

Figure S2 HPLC Chromatogram of (A) mixed solution consisting of glipizide (1), indomethacin (2), and celecoxib (3), (B) ligand fished by Fe_3O_4@C@PDA-Ni^{2+}@COX-2 and (C) residual solution after mixed fishing.

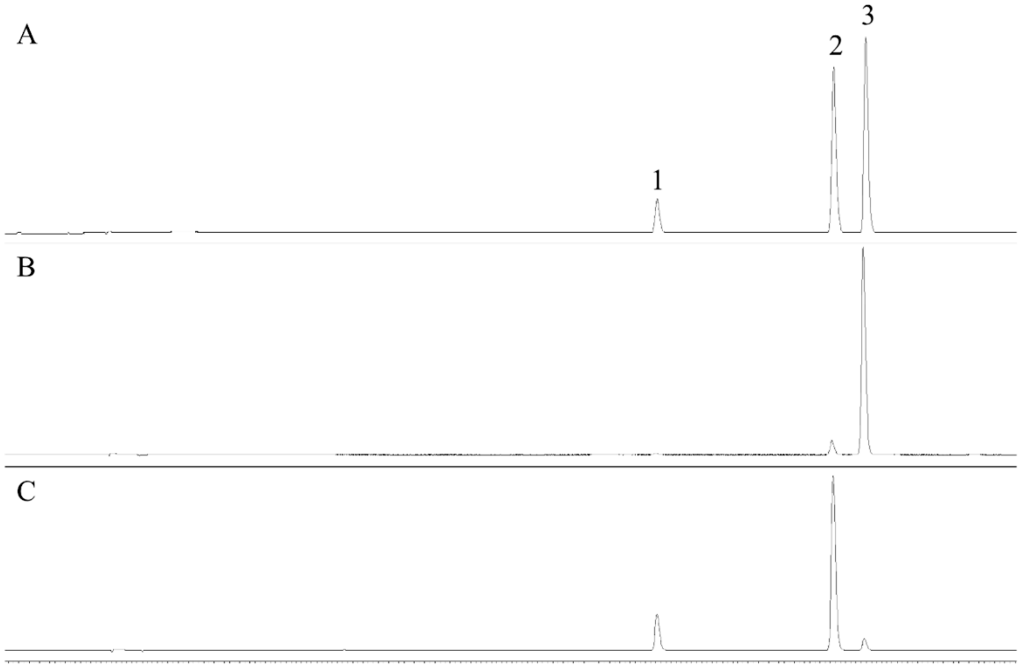

Figure S3 Total ion chromatograms of (A) *Panax quinquefolium* extract and (B) fishing liquid

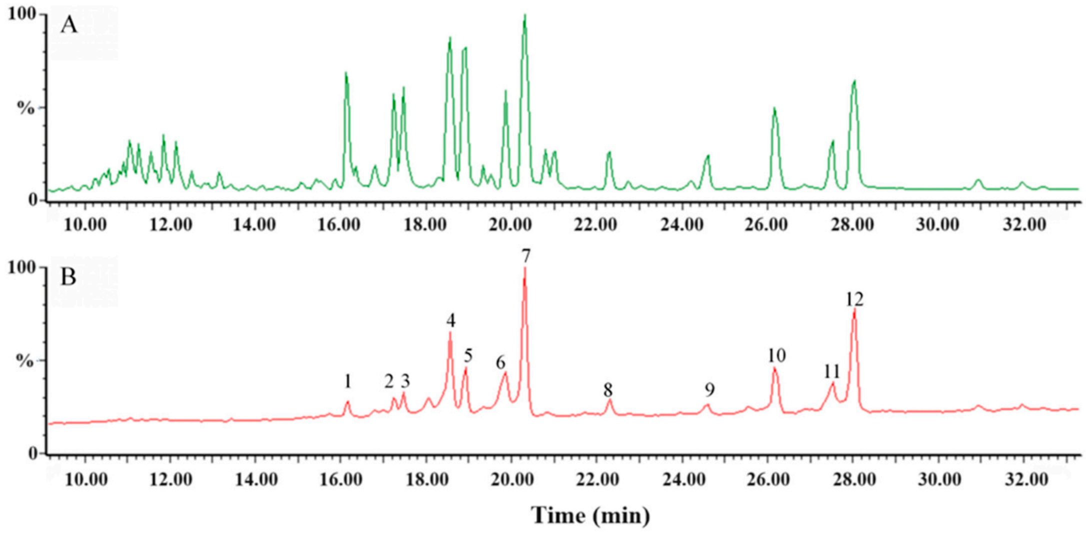

Figure S4 Structures of compounds 1-12

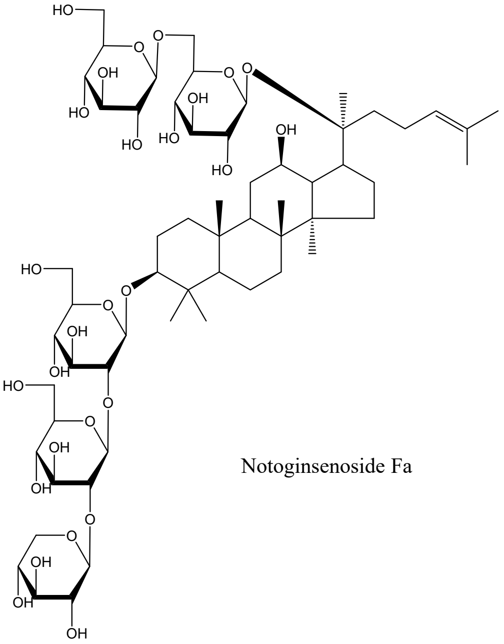

Notoginsenoside Fa

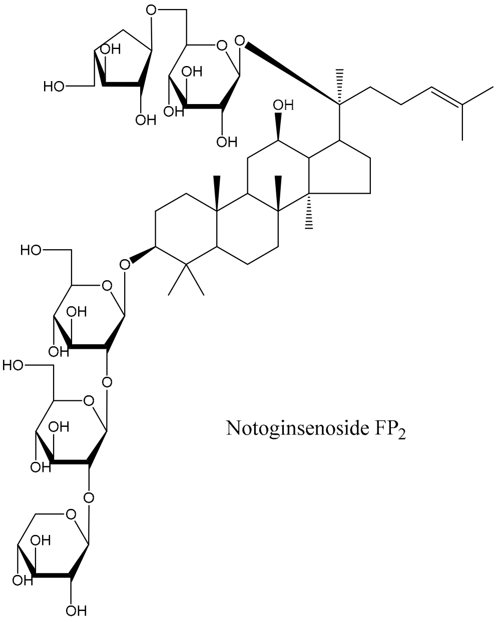

Notoginsenoside FP_2

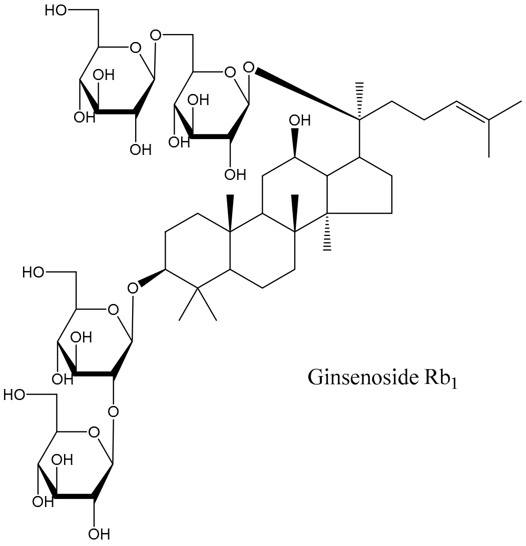

Ginsenoside Rb_1

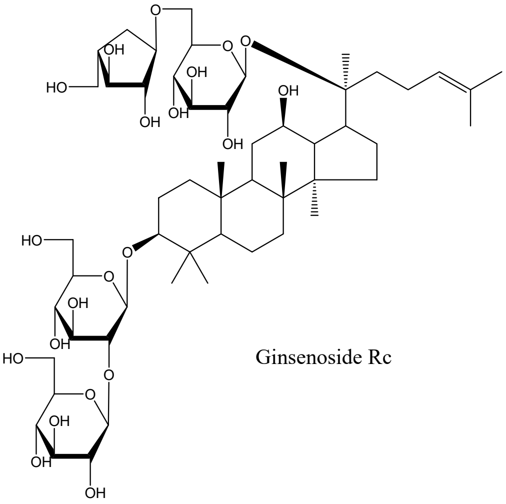

Ginsenoside Rc

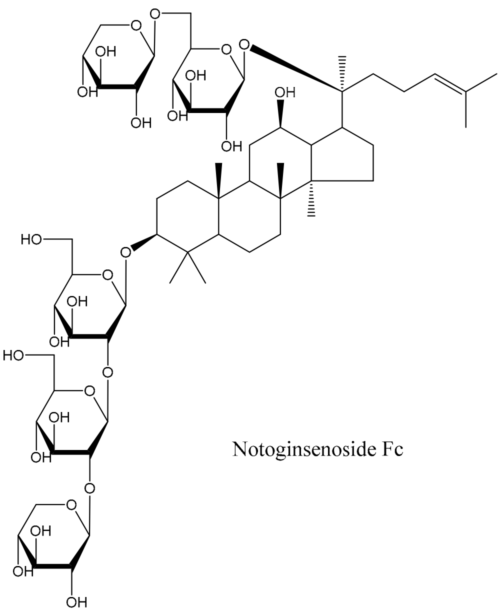

Notoginsenoside Fc

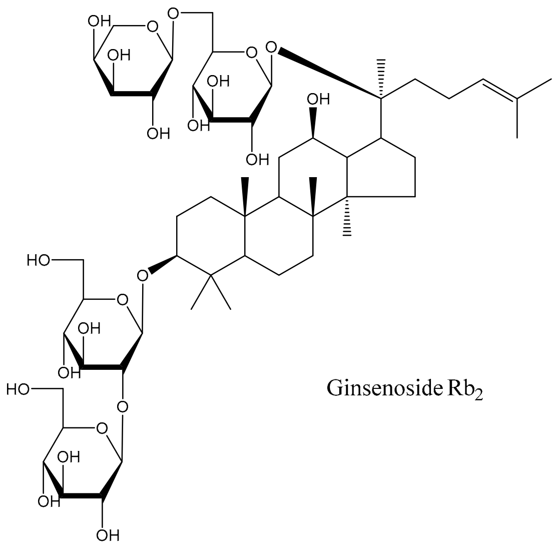

Ginsenoside Rb_2

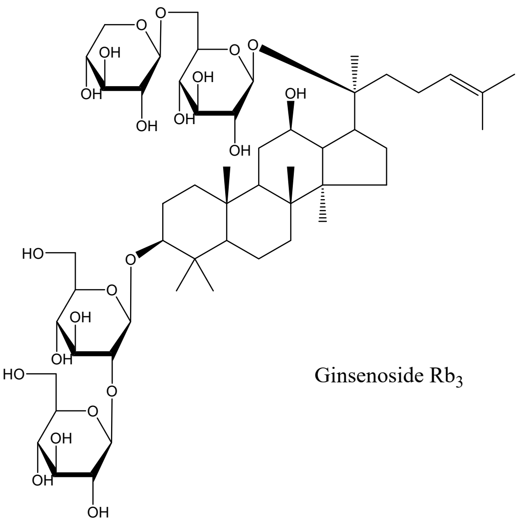

Ginsenoside Rb_3

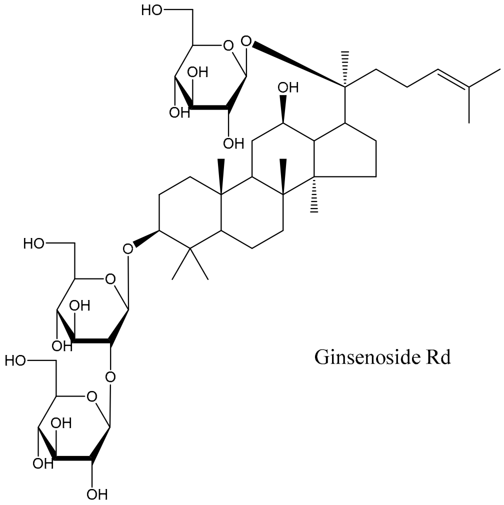

Ginsenoside Rd

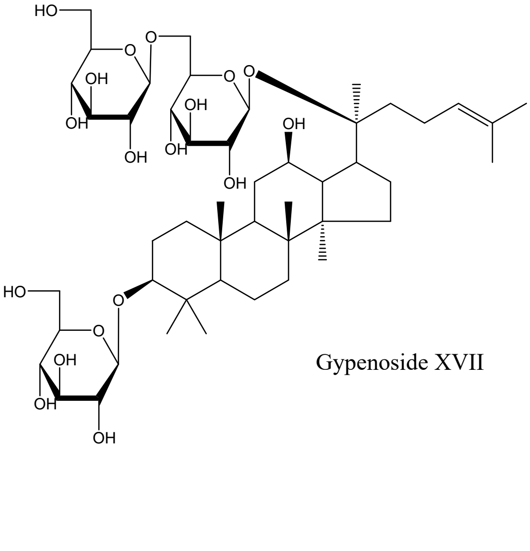

Gypenoside XVII

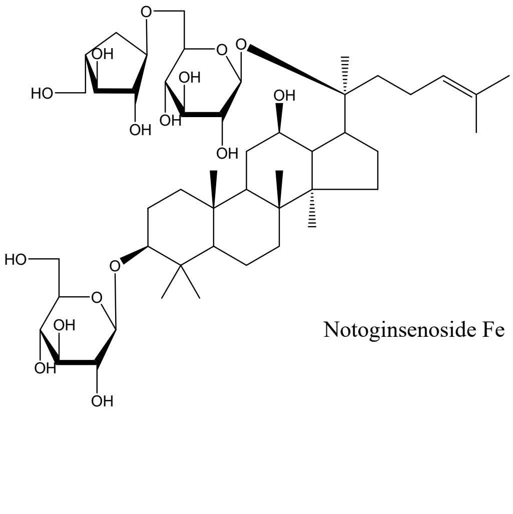

Notoginsenoside Fe

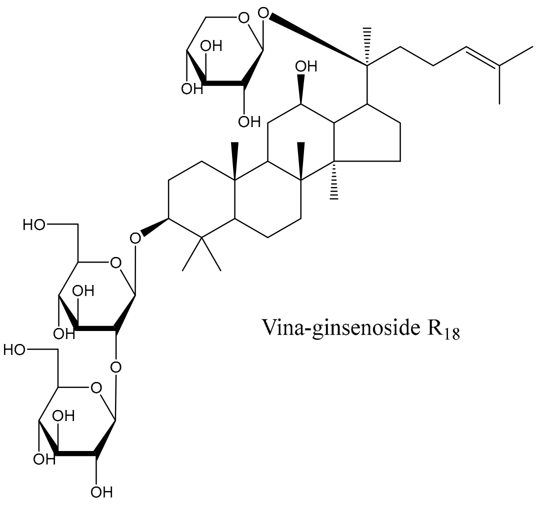

Vina-ginsenoside R_{18}

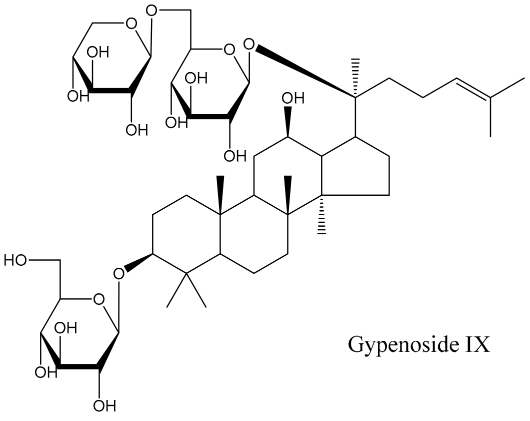

Gypenoside IX

Table S1 The atomic ratios of XPS analysis of Fe_3O_4@C@PDA-Ni^{2+}@COX-2

Name	Binding Energy/eV	Atomic/%
C1*s*	284.30	41.16
N1*s*	399.26	1.99
O1*s*	530.31	33.05
Fe2*p*	710.16	8.60
Ni2*p*	888.13	0.38

Table S2 Parameters of the pseudo-first-order kinetic model and pseudo-second-order kinetic model

model	parameters		model	parameters	
pseudo-first-order kinetic	k_1	0.04534607	pseudo-second-order kinetic	k_2	8.79844E-05
	q_e	0.002453502		q_e	106.6098081
	R^2	0.7988		R^2	0.9998

Table S3 Parameters of the Langmuir isothermal adsorption model and Freundlich isothermal adsorption model

model	parameters		model	parameters	
Langmuir isothermal adsorption	q_m	77.04160247	Freundlich isothermal adsorption	n	-8.04375804
	k_L	-13.03852996		k_f	3333114.954
	R^2	0.991		R^2	0.8325
